# Outcome and toxicity of stereotactic body radiotherapy with helical tomotherapy for inoperable lung tumor: analysis of Grade 5 radiation pneumonitis

**DOI:** 10.1093/jrr/rrt146

**Published:** 2014-01-23

**Authors:** Norihiro Aibe, Hideya Yamazaki, Satoaki Nakamura, Takuji Tsubokura, Kana Kobayashi, Naohiro Kodani, Takuya Nishimura, Haruumi Okabe, Kei Yamada

**Affiliations:** 1 Department of Radiology, Graduate School of Medical Science, Kyoto Prefectural University of Medicine, 465 Kajiicho Kawaramachi Hirokoji, Kamigyo-ku, Kyoto 602-8566, Japan; 2 Department of Radiology, Ujitakeda Hospital, Uji Satojiri 36-26, Uji City, Kyoto 611-0021, Japan

**Keywords:** lung cancer, SBRT, helical tomotherapy, severe radiation pneumonitis

## Abstract

To analyze outcomes and toxicities of stereotactic body radiotherapy with helical tomotherapy (HT-SBRT) for inoperable lung tumors, the medical records of 30 patients with 31 lung tumors treated with HT-SBRT were reviewed. The 3-year local control, cause-specific survival and overall survival rates (LC, CCS and OS, respectively) were analyzed using the Kaplan–Meier method. Toxicities were graded using Common Terminology Criteria for Adverse Events ver. 4. To investigate the factors associated with Grade 5 radiation pneumonitis (G5 RP), several parameters were analyzed: (i) patient-specific factors (age, gross tumor volume and PTV, and the interstitial pulmonary shadow on pretreatment CT); and (ii) dosimetry-specific factors (conformity index, homogeneity index, mean lung dose, and V5, V10, V15, V20 and V25 of the total lungs). The median duration of observation for all patients was 36.5 months (range, 4–67 months). The 3-year LC, CCS and OS were 82, 84 and 77%, respectively. Regarding Grade 3 or higher toxicities, two patients (6.7%) developed G5 RP. GTV was significantly associated with G5 RP (*P* = 0.025), and there were non-significant but slight associations with developing G5 RP for V5 (*P* = 0.067) and PTV (*P* = 0.096). HT-SBRT led to standard values of LC, CCS and OS, but also caused a markedly higher incidence of G5 RP. It is essential to optimize patient selection so as to avoid severe radiation pneumonitis in HT-SBRT.

## INTRODUCTION

Stereotactic body radiotherapy (SBRT) represents a paradigm shift in radiation therapy for surgically inoperable patients with early-stage non-small-cell lung cancer (NSCLC) and selected metastatic lung tumors [1]. Increasing evidence suggests that SBRT should be considered as a standard treatment option for medically inoperable lung tumors. A recent systematic review revealed that SBRT ensures consistent local control and overall survival rates comparable with surgical resection with acceptable, minimal side-effects, regardless of differences in dose-delivery modalities or dose-fractionation schedules, provided an adequate dose is delivered to the target [2].

Helical tomotherapy delivers intensity-modulated radiotherapy (IMRT) at a highly conformal dose to a target with a minimized dose to surrounding organs at risk (OARs). The onboard registration and image-guidance system, the megavoltage computed tomography (MVCT) scanner, offers precise real-time positional coordinates of a target or OAR to provide accurate daily set-up and target localization [3]. For hypofractional treatment such as SBRT for lung tumors, the helical tomotherapy unit has advantages but also causes some concerns: as advantages, conformal delivery by IMRT and precise set-up using the onboard MVCT system [4]; as concerns, expansion of the low-dose irradiated lung volume caused by the multidirectional and coplanar delivery method, and a discrepancy between calculated and delivered dose distributions due to movements of tumors and mechanical dynamics peculiar to helical tomotherapy: motions of the multileaf collimator, gantry rotation, and couch translation through the gantry. Despite these concerns, several experimental studies have shown that efforts to restrict respiration-associated tumor motion and optimize the planning target volume (PTV) margin lead to an acceptable minimal dose error [5–7], and a number of clinical reports on HT-SBRT have demonstrated its feasibility, with a promising outcome and favorable tolerance [4, 8–10].

Since March 2007, we have performed HT-SBRT for primary lung tumors, with a median follow-up duration of 36.5 months. In this study, we present outcomes and toxicities of HT-SBRT.

## MATERIALS AND METHODS

### Patient and tumor characteristics

Between March 2007 and February 2013, 31 primary lung tumors of 30 patients were treated with HT-SBRT. All patients provided written informed consent. The pretreatment characteristics of patients and tumors are summarized in Table 1 [11]. All patients were clinically diagnosed with primary lung cancer and considered medically inoperable because of chronic pulmonary or cardiovascular disease, an advanced age, or other complicating diseases. One patient had two separate nodules in the same lobe, in close proximity. Four patients were clinically diagnosed without pathologically confirmed malignancy because they refused or had contraindications to pathologic diagnosis.

**Table 1.** Pretreatment characteristics

Patient characteristics	*n* = 30
Age (year)	
Median (range)	80 (63–88)
Sex	
Male	20
Female	10
Clinical stage^a^	
cT1N0M0	25
cT2N0M0	4
cT3N0M0	1
PIPS grade	
Slight	24
Moderate	5
Severe	1
Tumor characteristics	*n* = 31
Histology	
Adenocarcinoma	19
Squamous cell carcinoma	5
NSCLC NOS	3
NA	4
Maximal diameter	
Median (range) (mm)	15 (6–36)
≤20 mm	23
21–30 mm	4
31–40 mm	4
GTV	
Median (range) (mm^3^)	3.8 (0.7–33.2)
Tumor location 1^b^	
Central	2
Peripheral	29
Tumor location 2	
Left upper lobe	8
Left lower lobe	7
Right upper lobe	11
Right middle lobe	2
Right lower lobe	3

PIPS = pretreatment interstitial pulmonary shadow, NSCLC NOS = non-small cell carcinoma not otherwise specified, NA = not available, GTV = gross tumor volume. ^a^Clinical stage decided according to 7th UICC TNM staging classification. ^b^Definition of tumor location 1 decided according to the report of Timmerman *et al.* [11].

### Treatment planning and dose delivery

All patients underwent three simulation CT scans (Aquilion 64 Toshiba Medical Co., Tokyo, Japan) under free breathing and during inhalation and exhalation phases under shallow breathing. The gross tumor volume (GTV) in each phase was delineated with the lung CT window setting (window level = −550 HU, width = 1600 HU) and a radiation treatment contouring system (FocalPro version 4.50, FOCAL, ELEKTA AB, Stockholm, Sweden). These GTVs of the three phases were fused and expanded as a PTV with a margin of 5–7 mm in transverse (5 mm in most cases) and 5–10 mm in longitudinal (5 mm in most cases) planes. The following structures were contoured as OARs: spinal cord, esophagus, lung and others as needed (heart, ipsilateral bronchus, liver, bowel, etc.). The lung volume on the dose–volume histogram (DVH) was defined as the bilateral lungs minus the combined volume of the three respiration-phase GTVs.

Treatment planning was conducted with the Tomotherapy Hi-Art System workstation (Tomotherapy Planning Station Version 3.1.5.3 Hi-Art system until December 2012, Tomotherapy Planning Station Version 4.2.1 Hi-Art system after December 2012; TomoTherapy Incorporated, Madison, WI, USA). Treatment was generally delivered so more than 90% (95% in most cases) of the PTV received the prescribed dose of 50 Gy, excluding the first patient, whose treatment was delivered so that 50% of the PTV received the prescribed dose. Dose constraints for OARs were based on the RTOG 0236 protocol [12]; this criterion was applied to 3-fraction treatment, so it was modified for use in 5-fraction treatment using the linear–quadratic formalism (biologically effective dose, BED) for convenience, although the linear–quadratic model generally overestimates the biological effect of high-dose-per-fraction radiotherapy [13–15]. The BED was calculated from *nd*[1 + *d*/(*α*/*β*)], where *n* is number of fractions, *d* is dose per fraction, and *α*/*β* is 2 Gy for the spinal cord and 3 Gy for the other organs to be avoided. Dose constraints are summarized in Table 2.

**Table 2.** Dose constraints for OARs

Lung (whole lung minus GTVs)	MLD	<10 Gy
	V20	<15%
Spinal cord	Any point	<22 Gy (4.4 Gy per fraction)
Esophagus or bowel	Any point	<33 Gy (6.6 Gy per fraction)
Heart	Any point	<37 Gy (7.4 Gy per fraction)
Trachea and ipsilateral bronchus	Any point	<37 Gy (7.4 Gy per fraction)
Ipsilateral brachial plexus	Any point	<30 Gy (6.0 Gy per fraction)

OAR = organ at risk, GTVs = volume combined with three gross tumor volumes on 3-phase simulation CT scans.

All patients were immobilized in a supine position with the BodyFix system (Body FIX^®^ Vacuum Pump P2, Medical Intelligence, Schwabműenchen, Germany) and underwent MVCT scanning for pretreatment set up with the tomotherapy registration system. Treatment was delivered after the patient and target were appropriately aligned three-dimensionally with the checkerboard and partial transparent image overlay to confirm that the target was within the PTV on the kVCT-MVCT-fusion image.

### Patient follow-up and evaluation

For the first year after treatment, follow-up evaluation and chest CT were conducted every 2–3 months; after the first year, 2–4 thoracic CT scans were taken every year. Post-treatment PET/CT scanning was performed as needed. Rates of local control, cause-specific, disease-free and overall survival (LC, CCS, DFS and OS, respectively) were evaluated. All adverse event severities were scored using the Common Terminology Criteria for Adverse Events (CTCAE) ver. 4.0.

We analyzed association between G5 RP and the following factors: (i) patient-specific factors (age, GTV, PTV and pretreatment interstitial pulmonary shadow (PIPS) on pretreatment CT); (ii) dosimetry-specific factors (conformity index (CI), homogeneity index (HI), mean lung dose (MLD), and V5, V10, V15, V20 and V25 of the bilateral lungs). The CI of one patient could not be obtained, so CI analysis was conducted with data from the remaining 29 patients. We defined PIPS as ground-glass opacity, linear or reticular shadows beneath the pleura, and honeycomb lung, and graded it as slight, moderate or severe based on the volume expressed as a percentage of the total lung volume as well as the grading system of Yamashita *et al*. [16]: slight, <10%; moderate, 10–50%; severe, >50%. CI was defined as the ratio of the treated volume (TV), which was enclosed by the prescription dose (PD) or 50 Gy, to the PTV volume (PTVV): CI = TV/PTVV. HI was defined as the ratio of the maximum dose in the PTV (Dmax) to the PD: HI = Dmax/PD. V*n* was defined as the lung volume receiving at least *n* Gy.

### Statistics

LC, DFS, CCS, OS and the incidence of G5 RP were calculated using the Kaplan–Meier method from the start of HT-SBRT. Mann–Whitney U or Fisher's exact tests were used to compare two independent groups of sampled data. All statistical calculations were conducted using StatView version 5.0 software (SAS Institute, Cary, NC, USA).

## RESULTS

All patients were treated successfully without complications. Median durations of observation for all patients and survivors were 36.5 (range, 4–67) and 38.5 (range, 7–67) months, respectively. Chemotherapy was not conducted for the duration of HT-SBRT. After HT-SBRT, chemotherapy was applied as maintenance therapy for one patient and salvage therapy for five with disease progression of primary lung cancers.

### Outcome (LC, DFS, CCS and OS) and toxicity

**Fig. 1.**
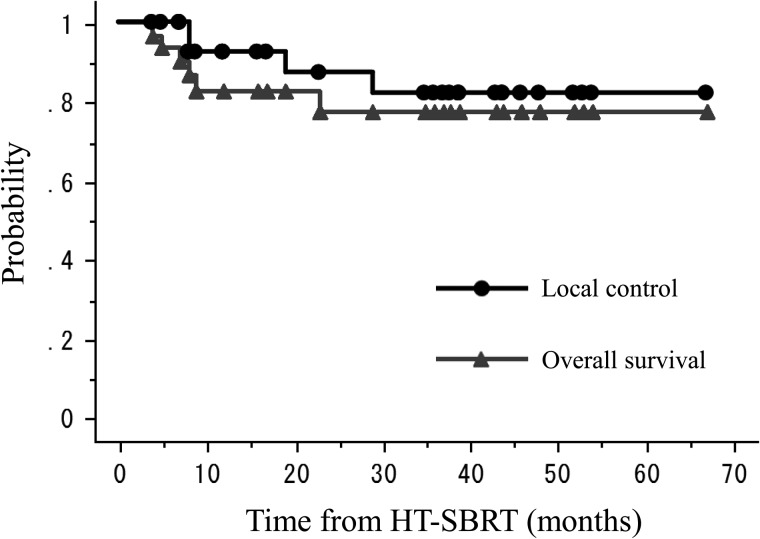
Kaplan-Meier actuarial local control and overall survival rates.

Up until the most recent follow-up, four patients had developed local recurrence, four suffered regional or distant recurrence, four died from their primary disease, and two died from other causes. One-, two- and three-year LC, DFS, CCS and OS were 93, 87 and 82%; 84, 69 and 69%; 90, 84 and 84%; and 83, 77 and 77%, respectively (Fig. 1).

**Fig. 2.**
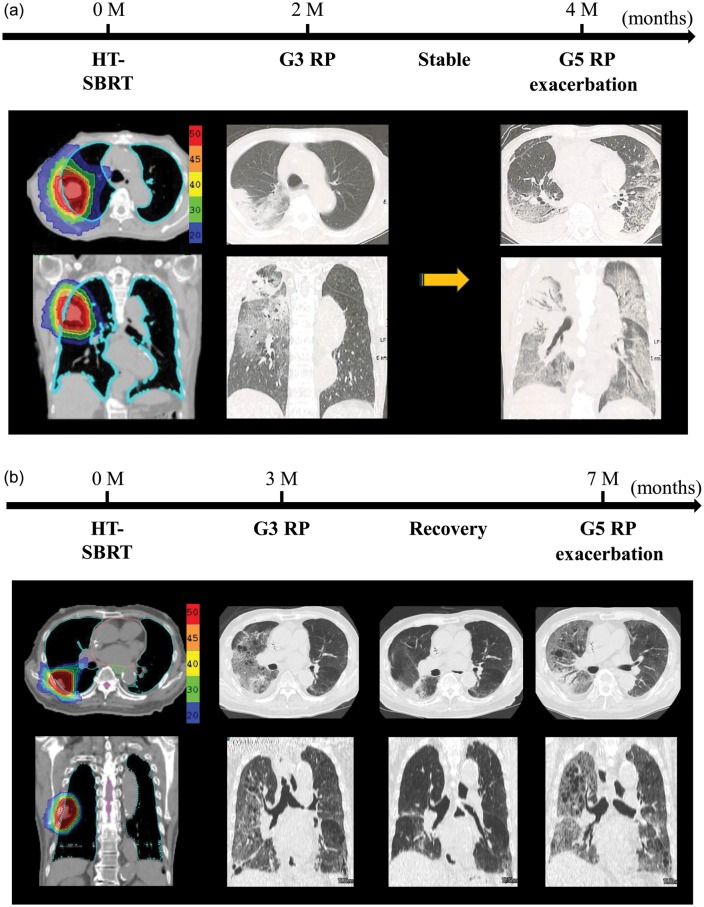
The time-series of the deterioration of radiation pneumonitis in two cases. Grade 3 radiation pneumonitis developed two and three months after HT-SBRT in Case 1 and Case 2, respectively. Steroid administration resolved the symptoms and pulmonary inflammatory shadow, but the relapse of radiation pneumonitis occurred after steroid discontinuation or tapering, resulting in the lethal exacerbation of radiation pneumonitis 4 and 7 months after HT-SBRT in Cases 1 and 2, respectively (**a**: Case 1; **b**: Case 2).

Regarding acute adverse events, mild radiation sickness developed in four cases. Subacute and late toxicities were as follows: radiation pneumonitis, hypoxia, atelectasis, pleural and cardiac effusion, chest pain, and rib fracture. Grade 2 or higher (G ≥ 2) toxicities are summarized in Table 3. For above Grade 2 toxicities, Grade 5 radiation pneumonitis (G5 RP) developed in two patients (Fig. 2). When they were diagnosed with symptomatic radiation pneumonitis 2–3 months after HT-SBRT (2 months in Case 1, 3 months in Case 2), the inflammatory changes on thoracic CTs were limited to the irradiated region. Although they were treated successfully with steroids and oxygenation, their radiation pneumonitis relapsed after the cessation or tapering of oral steroids. The relapsed radiation pneumonitis exacerbated rapidly and their pulmonary shadows expanded to the contralateral lung, resulting in death 4 and 7 months after HT-SBRT in Case 1 and 2, respectively. The cumulated incidence of G5 RP was 3.3% at 4 months, and 6.8% at 7 months after the start of HT-SBRT. An autopsy was performed in Case 1, revealing that the predominant pattern of bilateral lung injury was diffuse alveolar damage with bronchitis, although the lung tumor was pathologically absent.

**Table 3.** Grade ≥ 2 adverse events

	Grade 2	Grade 3	Grade 4	Grade 5
Radiation pneumonitis	1			2
Hypoxia	1			
Pleural effusion	1			
Pericardial effusion	2			

G ≥ 2 RP = Grade 2 or higher radiation pneumonitis.

### Analysis of G5 RP

Patient- and dosimetry-specific factors were analyzed to investigate the association with G5 RP. Table 4 shows these parameters according to the subgroup with G ≤ 2 and G > 2 RP, or G5 RP. When comparing these subgroups, the Mann–Whitney U test showed significance only for the GTV volume (*P* = 0.025). V5 and the PTV showed non-significant but slight associations with developing G5 RP (V5, *P* = 0.067; PTV, *P* = 0.096). When we compared the groups with or without G ≥ 2 RP, no significant differences were observed in any parameters including GTV, V5 and PTV (GTV, *P* = 0.14; V5, *P* = 0.32; PTV, *P* = 0.35).

**Table 4.** Analysis of risk factors correlated with G5 RP

		All patients		G ≤ 2 RP	G5 RP		G5 RP vs G ≤ 2 RP
		(*n* = 30)		(*n* = 28)	(*n* = 2)		
		Median	(range)	Median	Case 1	Case 2	*P-*value
Age	(year)	80	(63–88)	80	78	83	0.802
GTV	(mm^3^)	4.1	(0.7–33.2)	3.8	33.2	24.1	0.025
PTV	(mm^3^)	27.5	(10.1–120.8)	26.2	120.8	47.7	0.096
CI		1.12	(0.46–1.47 )	1.12	1.12	1.12	0.931
HI		1.06	(1.02–1.12 )	1.06	1.08	1.09	0.212
MLD	(Gy)	4.1	(1.9–8.7)	3.97	8.7	4.8	0.114
V5	(%)	21.4	(8.5–47.4)	20.5	43.6	25.5	0.067
V10	(%)	11.3	(5.4–25.9)	10.8	25.9	12.2	0.135
V15	(%)	7.3	(3.4–18.4)	6.9	18.4	7.5	0.183
V20	(%)	5.0	(2.0–13.9)	4.8	13.9	5.3	0.135
V25	(%)	3.8	(1.5–10.6)	3.7	10.6	3.9	0.157

G5 RP = Grade 5 radiation pneumonitis, G ≤ 2 RP = Grade 2 or lower radiation pneumonitis, GTV = gross tumor volume, PTV = planning target volume, CI = conformity index, HI = homogeneity index, MLD = mean lung dose, V*n* = lung volume (%) receiving at least *n* Gy of radiation.

For PIPS, all patients were graded as follows: 24 patients, ‘slight’; 5 patients, ‘moderate’; 1 patient, ‘severe’. In the ‘slight’ and ‘moderate’ groups, one patient developed G5 RP, respectively, so the incidence of G5 RP was 4.2% in the ‘slight’ and 16.7% in the higher grade (‘moderate’ and ‘severe’) groups, but this was non-significant using Fisher's exact test (*P* = 0.37).

## DISCUSSION

This retrospective analysis demonstrated that HT-SBRT led to a 3-year LC, CCS and OS of 82, 84 and 77%, respectively and caused G5 RP in two cases. Our analysis for G5 RP showed the GTV volume was significantly associated.

Many studies of linac-based SBRT for early-stage NSCLC have demonstrated favorable LC, CCS and OS comparable with surgery with acceptable adverse effects. A recent systematic review showed that SBRT led to promising outcomes: median 1-, 2- and 3-year LC were 92, 87 and 81%; median 1-, 2- and 3-year CCS were 94, 77 and 72%; and median 1-, 2- and 3-year OS were 83, 65 and 58%, respectively [2]. Although the follow-up duration differed, our results are comparable with the median values of this review. Along with this study, Hodge *et al*. [4], Baisden *et al*. [8] and Tomita *et al*. [9] reported excellent LC with HT-SBRT, although their follow-up durations were shorter. These results suggest that HT-SBRT can achieve disease control comparable with SBRT with other linac-based modalities, although more clinical data and a longer follow-up are needed to evaluate HT-SBRT.

For radiation pneumonitis, in our series, HT-SBRT caused G2 RP in one case and G5 RP in two cases. Several investigators reported that the incidence of G ≥ 2 RP is ∼5–20% in lung SBRT [17–21]. Therefore, our incidence (10%) of G ≥ 2 RP is similar to that of previous studies. However, the incidence (6.7%) of G5 RP in this series is markedly higher than in previous reports. In the above review, only six studies reported the development of G5 toxicities among the 55 analyzed [2].

To explore the causes of G5 RP in this series, we analyzed the relationship between G5 RP and patient- or dosimetry-specific factors. It is true that G5 RP can be caused not only by the factors we investigated, but also by those we did not: comorbidity, the patient's condition at RP diagnosis, medication for RP, etc. Indeed, our two patients who suffered from G5 RP had many pretreatment complications: Case 1 had a history of coronary-artery bypass graft surgery and hemodialysis for chronic renal failure; Case 2 had pulmonary and cardiovascular dysfunction and suspected idiopathic interstitial pneumonitis. Therefore, it was difficult to identify a clear factor associated with G5 RP in this analysis. However, in this series, the GTV was a significant parameter (*P* = 0.025).

**Fig. 3.**
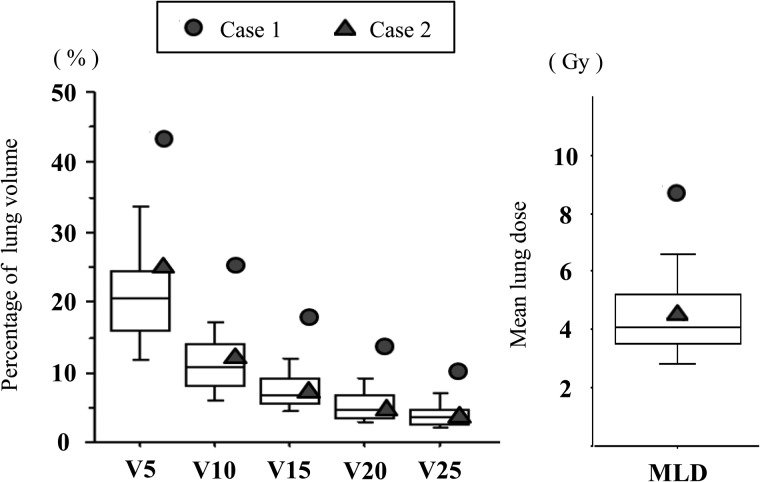
V5–V25 and MLD of all patients. A dose–volume histogram (V5–V25) and the mean lung dose (MLD) of the normal lung volume in all patients. The box includes the central 50% of data (25–75%), and the central 80% of data (10–90%) are contained within the error bars. The solid line within each box indicates the median of the data. Circles (solid symbol) show the data of Case 1, and triangles (solid symbol) show those of Case 2. V*n* = volume of lung (%) receiving ≥*n* Gy.

Generally, the GTV is proportional to the PTV, and the irradiated lung volume increases with a target volume increase [22]. However, we found a significant difference only in the GTV and not the PTV. This may have resulted from the low statistical power of the small sample size, so we believe that a larger target volume of the GTV or the PTV is strongly associated with severe RP. Indeed, several lung SBRT studies with a larger sample size have reported the target volume as one of the significant predictors of symptomatic RP. Baker *et al*. [20] reported the tumor volume as a significant predictor of symptomatic RP. Matsuo *et al*. [18] revealed that the symptomatic RP rate was significantly higher in patients with a PTV ≥ 37.7 mm^3^, compared with those with a smaller volume (34.5 vs 11.1%, respectively). Their analysis of three subgroups (patients with a PTV < 37.7 mm^3^; those with a PTV ≥ 37.7 mm^3^ and V25 < 4.2%; and those with a PTV ≥ 37.7 mm^3^ and V25 ≥ 4.2%) showed that the incidences of G2 ≥ RP increased with elevated PTV (11.1, 23.5 and 50%, respectively). Ong *et al*. [23] reported a high RP risk in patients with a PTV exceeding 80 mm^3^. Their retrospective analysis showed that, in 18 patients with a PTV ≥ 80 mm^3^, two deaths were potentially treatment-related and 5 patients developed symptomatic radiation pneumonitis at a median follow-up of 12.8 months, concluding that patients with a large PTV exceeding 80 mm^3^ had a higher risk of radiation pneumonitis. Table 4 and Fig. 3 show that the target volume of Case 1 was the largest among all patients (GTV, 33.2 mm^3^; PTV, 120.8 mm^3^), resulting in the largest V10–V25 volume and MLD. Therefore, G5 RP may have been mainly due to the large target volume.

**Fig. 4.**
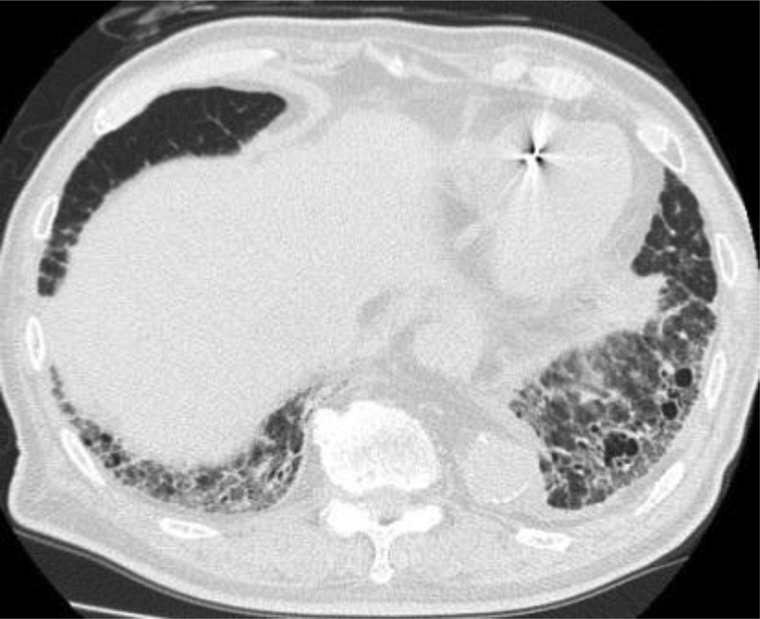
The pretreatment interstitial shadow of Case 2.

In Case 2, the GTV (24.1 mm^3^) and the PTV (47.7 mm^3^) were not large. Indeed, PTV was similar to the median value (48 mm^3^) in the large series of 251 patients reported by Barriger *et al.* [21], who described an overall rate of G2–4 RP of 9.4% and no G5 RP case. Furthermore, in Case 2, V5–V25 and MLD values were slightly higher than the medians of our series, making it difficult to explain the onset or deterioration to G5 RP solely using the target size and irradiated lung volume. Based on the results of Matsuo *et al*. [18], we could predict that Case 2 had a relatively high risk (23.5%) of G ≥ 2 RP because the PTV and V25 values were 47.7 cm^3^ and 3.9%, respectively. However, their analysis was based on the incidence of G ≤ 3 RP, and we could not clarify why RP of Case 2 aggravated to G5 RP. Thus, it was difficult to explain the cause of G5 RP in Case 2 solely based on dosimetric parameters, but his poor pretreatment pulmonary condition may have been a factor causing the onset or deterioration to severe RP. Case 2 had a history of suspected idiopathic interstitial pneumonitis and moderate PIPS (Fig. 4). Yamashita *et al*. [16] reported that the incidence of severe RP (Grade 4 or higher) was significantly correlated with a shadow of interstitial pneumonitis on pretreatment CT, which they defined as a mandatory observation beneath the pleura and honeycomb lung, as well as higher pretreatment values of serum KL-6 (Krebs von den Lungen-6) and SP-D (surfactant protein-D), the upper limits of which were defined as 500 U/mm^3^ and 110 ng/mm^3^, respectively. Although data on the serum biomarkers KL-6 and SP-D before HT-SBRT were unavailable in our study, our analysis of PIPS showed a non-significant but 4-fold absolute difference between the incidence of G5 RP in the ‘slight’ and ‘moderate and severe’ groups (4.2 vs 16.7%, respectively, *P* = 0.37). The limited size of our series may have led to insufficient statistical power to analyze the impact of PIPS on severe RP. Thus, in Case 2, there was no clear cause of G5 RP; however, the moderate PIPS may have been related to the onset or deterioration to severe RP. Yamashita *et al*. [16] succeeded in reducing severe RP by patient selection for lung SBRT, whereby patients were excluded if they had a clear IP shadow on pretreatment CT or if serum KL-6 and SP-D levels were high. Their selection criteria may be useful to avoid severe radiation pneumonitis.

Our study had several limitations. First, it was retrospective, so the presence of a bias cannot be completely excluded. Second, total numbers of patients and events were low, limiting statistical power and accuracy. Third, the factors investigated might not be sufficient to explain severe RP. In addition, the effect of the HT-SBRT itself could not be directly analyzed because all patients were treated with HT-SBRT. Although this study had such limitations, it identified a significant impact of the target volume on severe RP, as well as a non-significant but higher incidence of G5 RP in the subgroup with moderate or severe PIPS, consistent with several previous reports. We believe that the development of severe RP or deterioration of moderate radiation pneumonitis to severe RP is closely associated with patient-specific parameters such as the target volume or PIPS, and that appropriate patient selection is essential to avoid severe RP in HT-SBRT. Further efforts to identify other factors causing radiation pneumonitis in HT-SBRT are needed to avoid critical toxicities.

## FUNDING

This work was not supported by any sources.

## References

[1] Videtic GM, Stephans KL (2010). The role of stereotactic body radiotherapy in the management of non-small cell lung cancer: an emerging standard for the medically inoperable patient?. Curr Oncol Rep.

[2] Chi A, Liao Z, Nguyen NP (2010). Systemic review of the patterns of failure following stereotactic body radiation therapy in early-stage non-small-cell lung cancer: clinical implications. Radiother Oncol.

[3] Welsh JS, Patel RR, Ritter MA (2002). Helical tomotherapy: an innovative technology and approach to radiation therapy. Technol Cancer Res Treat.

[4] Hodge W, Tome WA, Jaradat HA (2006). Feasibility report of image guided stereotactic body radiotherapy (IG-SBRT) with tomotherapy for early stage medically inoperable lung cancer using extreme hypofractionation. Acta Oncol.

[5] Klein M, Gaede S, Yartsev S (2013). A study of longitudinal tumor motion in helical tomotherapy using a cylindrical phantom. J Appl Clin Med Phys.

[6] Kanagaki B, Read PW, Molloy JA (2007). A motion phantom study on helical tomotherapy: the dosimetric impacts of delivery technique and motion. Phys Med Biol.

[7] Kissick MW, Flynn RT, Westerly DC (2008). On the impact of longitudinal breathing motion randomness for tomotherapy delivery. Phys Med Biol.

[8] Baisden JM, Romney DA, Reish AG (2007). Dose as a function of lung volume and planned treatment volume in helical tomotherapy intensity-modulated radiation therapy-based stereotactic body radiation therapy for small lung tumors. Int J Radiat Oncol Biol Phys.

[9] Tomita N, Kodaira T, Matsuo M (2010). Helical tomotherapy for solitary lung tumor: feasibility study and dosimetric evaluation of treatment plans. Technol Cancer Res Treat.

[10] Chi A, Jang SY, Welsh JS (2011). Feasibility of helical tomotherapy in stereotactic body radiation therapy for centrally located early stage nonsmall-cell lung cancer or lung metastases. Int J Radiat Oncol Biol Phys.

[11] Timmerman R, McGarry R, Yiannoutsos C (2006). Excessive toxicity when treating central tumors in a phase II study of stereotactic body radiation therapy for medically inoperable early-stage lung cancer. J Clin Oncol.

[12] RTOG 0236 Protocol Information A phase II trial of stereotactic body radiation therapy (SBRT) in the treatment of patients with medically inoperable stage I/II non-small cell lung cancer. http://www.rtog.org/ClinicalTrials/ProtocolTable/StudyDetails.aspx?study=0236.

[13] Shibamoto Y, Otsuka S, Iwata H (2012). Radiobiological evaluation of the radiation dose as used in high-precision radiotherapy: effect of prolonged delivery time and applicability of the linear-quadratic model. J Radiat Res.

[14] Iwata H, Matsufuji N, Toshito T (2013). Compatibility of the repairable-conditionally repairable, multi-target and linear-quadratic models in converting hypofractionated radiation doses to single doses. J Radiat Res.

[15] Wada M, Suzuki M, Liu C (2013). Modeling the biological response of normal human cells, including repair processes, to fractionated carbon beam irradiation. J Radiat Res.

[16] Yamashita H, Kobayashi-Shibata S, Terahara A (2010). Prescreening based on the presence of CT-scan abnormalities and biomarkers (KL-6 and SP-D) may reduce severe radiation pneumonitis after stereotactic radiotherapy. Radiat Oncol.

[17] Takeda A, Sanuki N, Kunieda E (2009). Stereotactic body radiotherapy for primary lung cancer at a dose of 50 Gy total in five fractions to the periphery of the planning target volume calculated using a superposition algorithm. Int J Radiat Oncol Biol Phys.

[18] Matsuo Y, Shibuya K, Nakamura M (2012). Dose–volume metrics associated with radiation pneumonitis after stereotactic body radiation therapy for lung cancer. Int J Radiat Oncol Biol Phys.

[19] Inoue T, Katoh N, Onimaru R (2013). Stereotactic body radiotherapy using gated radiotherapy with real-time tumor-tracking for stage I non-small cell lung cancer. Radiat Oncol.

[20] Baker R, Han G, Sarangkasiri S (2013). Clinical and dosimetric predictors of radiation pneumonitis in a large series of patients treated with stereotactic body radiation therapy to the lung. Int J Radiat Oncol Biol Phys.

[21] Barriger RB, Forquer JA, Brabham JG (2010). A dose-volume analysis of radiation pneumonitis in non-small cell lung cancer patients treated with stereotactic body radiation therapy. Int J Radiat Oncol Biol Phys.

[22] Takeda A, Kunieda E, Sanuki N (2009). Dose distribution analysis in stereotactic body radiotherapy using dynamic conformal multiple arc therapy. Int J Radiat Oncol Biol Phys.

[23] Ong CL, Palma D, Verbakel WF (2010). Treatment of large stage I–II lung tumors using stereotactic body radiotherapy (SBRT): planning considerations and early toxicity. Radiother Oncol.

